# Generalization Bounds for Coregularized Multiple Kernel Learning

**DOI:** 10.1155/2018/1853517

**Published:** 2018-11-01

**Authors:** Xinxing Wu, Guosheng Hu

**Affiliations:** Department of Communication and Information Engineering, Shanghai Technical Institute of Electronics & Information, Shanghai 201411, China

## Abstract

Multiple kernel learning (MKL) as an approach to automated kernel selection plays an important role in machine learning. Some learning theories have been built to analyze the generalization of multiple kernel learning. However, less work has been studied on multiple kernel learning in the framework of semisupervised learning. In this paper, we analyze the generalization of multiple kernel learning in the framework of semisupervised multiview learning. We apply Rademacher chaos complexity to control the performance of the candidate class of coregularized multiple kernels and obtain the generalization error bound of coregularized multiple kernel learning. Furthermore, we show that the existing results about multiple kennel learning and coregularized kernel learning can be regarded as the special cases of our main results in this paper.

## 1. Introduction

Kernel-based learning is related to achieve nonlinear machine learning tasks from linear ones. In the real applications, selecting a good or suitable kernel for the kernel-based learning is an important and difficult task. To this end, an approach named multiple kernel learning has been developed, and it allows to automatically choose the best kernel from a predefined kernel class. The earliest work of multiple kernel learning can be traced back to the research in [1], where the authors proposed to automatically pick up a linear combination of candidate kernels for the support vector machines based on a semidefinite programming approach. Theoretical generalization analysis of multiple kernel learning has been widely studied by many researchers [1–7]. In particular, Ying and Campbell in [2] proposed a novel generalization bound (Rademacher chaos complexity) for the study of multiple kernel learning. However, the discussions in [2] were for single view and supervised learning. In this paper, we will employ Rademacher chaos complexity proposed in [2] to study the generalization error of coregularized multiple kernel learning in the semisupervised multiview learning framework.

Semisupervised multiview learning as an area of machine learning is trained with both labeled samples and unlabeled samples, and the unlabeled samples are helpful to reduce the amount of the labeled samples. Semisupervised multiview learning supposes that the train samples can be represented by multiple views. The coregularized least squares algorithm—a semisupervised version of regularized least squares with two views—is a typical multiview learning model that uses the unlabeled samples to estimate the view incompatibility of models [8, 9]. Rosenberg in [10] extended the coregularized least squares algorithm to the case of kernel cotraining. And Brefeld et al. and Rosenberg in [11, 12, 13] discussed the generalization bound of kernel-based learning with multiple (or two) views in the semisupervised learning framework. However, the discussions in [11, 12, 13] supposed that the kernel used to construct the reproducing kernel Hilbert space is predefined. Therefore, their results cannot be used to the analysis of multiple kernel learning.

In this paper, we will discuss the generalization error of coregularized multiple kernel learning in the semisupervised multiview learning framework. And we show that the results in [2] and [11] can be regarded as the special cases of our main results. The rest of the paper is organized as follows. In Section 2, we introduce some basic notations and definitions for later discussion. In Section 3, we discuss the related research and put forward the question that will be studied in this paper. In Section 4, we present our main results. In Section 5, we give the main proofs for our main results proposed in Section 4. In Section 6, we give a comparative discussion of our results to the existing work and show that the results about multiple kennel learning in [2] and coregularized kernel learning in [11] can be regarded as the special cases of our main results. The last Section 7 concludes this paper.

## 2. Notations and Definitions

In this section, we introduce notations and definitions for later discussions:

- Let *ℕ* be the set of natural numbers and *ℝ* be the set of real numbers. Let *ℕ*_*n*_={1,2,…, *n*}, *n* ∈ *ℕ*.
- Let (Ω, *𝒜*, P) be a probability space; that is, Ω alone is called the sample space, *𝒜* is a *σ*-algebra on Ω, and P is a probability measure on (Ω, *𝒜*). And Ω has the structure Ω=*𝒳* × *𝒴*(⊂*ℝ*), where *𝒳* and *𝒴* are the input space and output space, respectively. Denote P_*𝒳*_ as the marginal distribution on *𝒳*. For ignoring the discussion of measure theory, we simply denote (Ω, *𝒜*, P) as (Ω, P).
- Let ℱ be the set of all measurable functions *f* : *𝒳*⟶*𝒴*. Assume that ℋ is a subset of ℱ. That is, ℋ ⊂ ℱ, the set ℋ is called the hypothesis class.
- Let *S*={*z*_*i*_=(*x*_*i*_, *y*_*i*_), *i* ∈ *ℕ*_*n*_} be a finite set of the labeled training samples, and assume these samples are independent and identically distributed (i.i.d.) according to P. Denote the bold letter as a vector; for example, **z** presents a vector (*z*_1_, *z*_2_,…, *z*_*n*_).
- For the sign |·|, if *D* is a set, we use |*D*| to represent the number of elements of a set and if *D* is a function, we use |*D*| to represent the absolute value of the function *D*.
- If *A* is a matrix, we use *A*^**T**^ to represent the transpose of the matrix *A*.
- Let *L* be the loss function, *L* : *𝒴* × *𝒴*⟶[0, +*∞*), and the loss of *f* on a sample point *z*=(*x*, *y*) is defined by *L*(*f*, *z*) or *L*(*f*(*x*), *y*).

In learning theory, one of the purposes is to pick up a function *f* in hypothesis space ℋ that minimizes the following generalization error:

(1) $$\begin{array}{c} E_{\text{P}}\left[L\left(f\left(X\right),Y\right)\right]≜\int_{\Omega }L\left(f,z\right)\;d\text{P}. \end{array}$$

Generally speaking, the distribution P in the above Equation (1) is unknown. Rather than minimizing *E*_P_[*L*(*f*(*X*), *Y*)], we usually minimize the empirical or training error below:

(2) $$\begin{array}{c} E_{n}\left(f,S\right)≜\frac{1}{m}\underset{i\in N_{m}}{\sum }L\left(f,z_{i}\right), \end{array}$$

where the sign *S* represents the finite labeled samples and *z*_*i*_=(*x*_*i*_, *y*_*i*_) ∈ *S*, *i* ∈ *ℕ*_*m*_.

In this paper, the main quantity we are interested in is the following uniform estimation of the difference between the generalization error and empirical error:

(3) $$\begin{array}{c} \underset{f}{\sup}\left\{E_{\text{P}}\left[L\left(f\left(X\right),Y\right)\right]-E_{n}\left(f,S\right)\right\}. \end{array}$$

For the discussion in the later sections, we introduce the following four definitions and one lemma (Definition 5 is proposed by us).

*Definition 1 (Empirical Rademacher Complexity) [2]*. Let ℋ be a class of functions *f* : Ω⟶*ℝ*. The samples *x*_*i*_, *i* ∈ *ℕ*_*n*_, are independently drawn from the probability space (Ω, P). The empirical Rademacher complexity can be defined as

(4) $$\begin{array}{c} {ℛ}_{n}\left(ℋ\right)≜E_{\sigma }\left[\underset{f\in ℋ}{\sup}\frac{1}{\sqrt{n}}\underset{i\in N_{n}}{\sum }\sigma_{i}f\left(x_{i}\right)\right], \end{array}$$

where the random variables *σ*_*i*_, *i* ∈ *ℕ*_*n*_, are Rademacher variables, and **σ** presents a vector (*σ*_1_, *σ*_2_,…, *σ*_*n*_).

*Definition 2 (Empirical Rademacher Chaos Complexity) [2]*. Let ℋ be a class of functions *f* : Ω×Ω⟶*ℝ*. The samples *x*_*i*_, *i* ∈ *ℕ*_*n*_, are independently drawn from the probability space (Ω, P). The empirical Rademacher chaos complexity can be defined as

(5) $$\begin{array}{c} U_{n}\left(ℋ\right)≜E_{\sigma }\left[\underset{f\in ℋ}{\sup}\frac{1}{n}\underset{i,j\in N_{n},i<j}{\sum }\sigma_{i}\sigma_{j}f\left(x_{i},x_{j}\right)\right], \end{array}$$

where the random variables *σ*_*i*_, *i* ∈ *ℕ*_*n*_, are Rademacher variables, and **σ** presents a vector (*σ*_1_, *σ*_2_,…, *σ*_*n*_).

*Definition 3 (Reproducing Kernel Hilbert Space, RKHS) [14]*. The function

(6) $$\begin{array}{c} K:X\times X⟶R, \end{array}$$

is a reproducing kernel of the Hilbert space ℋ_*K*_ if and only if.

- For any *x* ∈ *𝒳*, *K*(·, *x*) ∈ ℋ_*K*_
- For any *f* ∈ ℋ_*K*_, for any *x* ∈ *𝒳*, 〈*f*, *K*(*x*)〉_ℋ_*K*__=*f*(*x*)

A Hilbert space of functions which possesses a reproducing kernel is called a reproducing kernel Hilbert space.

***Remark 1*.**

The second condition in the above Definition 3 is called “the reproducing property”: the value of the function *f* at the point *x* is reproduced by the inner product of *f* with *K*(*x*). From the above two conditions, for any, (*x*, *y*) ∈ *𝒳* × *𝒳*, it is clear that

(7) $$\begin{array}{c} K\left(x,y\right)={\langle K\left(x,\cdot \right),K\left(y,\cdot \right)\rangle }_{{ℋ}_{K}}. \end{array}$$

In real applications, the solution to many reproducing kernel Hilbert space optimization questions is contained in a special subspace of the reproducing kernel Hilbert space, often known as the “span of the data”. The span of the data for an reproducing kernel Hilbert space ℋ_*K*_ is the linear subspace (Appendix A.3 on page 75 of [13]):

(8) $$\begin{array}{c} \text{span}\left\{K\left(x_{i},\cdot \right):x_{i}\in X,i\in N_{m}\right\}. \end{array}$$

For simplicity, we denote the above linear subspace by ℒ_*m*_^*K*^.

Let *𝒦* ⊂ {*K* : *𝒳* × *𝒳*⟶*ℝ*} be a class of kernels. In this paper, we assume that $\kappa ≜\sup_{K\in 𝒦,x\in 𝒳}\sqrt{K\left(x,x\right)}$ is finite.

*Definition 4 (Subnormalized Functional with Degenerate Dimension n)*. If a loss function *ℓ* : (∏_*i*∈*ℕ*_*n*__ℋ_*K*_*i*__) × *𝒴*⟶[0, +*∞*) satisfies

(9) $$\begin{array}{c} \ell \left(\underset{n}{\underset{︸}{0,\ldots ,0}},y\right)\leq 1,\text{for any }y\in Y, \end{array}$$

then we call *ℓ* a subnormalized functional with degenerate dimension *n* on (∏_*i*∈*ℕ*_*n*__ℋ_*K*_*i*__) × *𝒴*.

**Lemma 1 .**

[13] Let ℋ be a reproducing kernel Hilbert space with kernel *K* : *𝒳* × *𝒳*⟶*ℝ*, and consider any point *x* ∈ *𝒳*. If ℒ^*K*^ is a closed subspace containing *K*(*x*), then the projection of f onto ℒ^*K*^ has the same value at x as f does. That is,

(10) $$\begin{array}{c} f\left(x\right)=\left({\text{Proj}}_{{ℒ}^{K}}f\right)\left(x\right). \end{array}$$

For multiple kernel learning, the main task is to automatically choose a kernel K from a predefined class *𝒦* of kernels, and find a function from the reproducing kernel Hilbert space ℋ_*K*_ that is most suitable to model the given samples.

In this paper, our purpose is to minimize

(11) $$\begin{array}{c} Q\left(f^{1},f^{2}\right)≜\frac{1}{m}\underset{i\in N_{m}}{\sum }\ell \left(f^{1}\left(x_{i}\right),f^{2}\left(x_{i}\right),y_{i}\right)+\gamma_{1}{\left|\left|f^{1}\right|\right|}_{{ℋ}_{K_{1}}}^{2}+\gamma_{2}{\left|\left|f^{2}\right|\right|}_{{ℋ}_{K_{2}}}^{2}+\lambda \overset{m+u}{\underset{i=m+1}{\sum }}{\left(f^{1}\left(x_{i}\right)-f^{2}\left(x_{i}\right)\right)}^{2}, \end{array}$$

over the class (∪_*K*_1_∈*𝒦*_ℋ_*K*_1__) × (∪_*K*_2_∈*𝒦*′_ℋ_*K*_2__). Here, let *𝒦* and *𝒦*′ be the classes of kernels. ℋ_*K*_1__ and ℋ_*K*_2__ denote the reproducing kernel Hilbert spaces. m represents the amount of the labeled points (*x*_*i*_, *y*_*i*_) ∈ *𝒳* × *𝒴*, *i* ∈ *ℕ*_*m*_, and u represents the amount of the unlabeled points *x*_*m*+*i*_ ∈ *𝒳*, *i* ∈ *ℕ*_*u*_. The signs *γ*_1_, *γ*_2_, and λ mean the regularization parameters. The functions *f*^1^ and *f*^2^, respectively, represent two viewers, and the function *ℓ*(*f*^1^(·), *f*^2^(·), ·) is the labeled loss function, which measures the performance of *f*^1^ and *f*^2^ on the labeled points *z*_*i*_, *i* ∈ *ℕ*_*m*_.

## 3. Related Work

In [11], Rosenberg and Bartlett used Rademacher complexity to bound the coregularized kernel class in the semisupervised two-view learning framework, and two viewers are two predefined reproducing kernel Hilbert spaces (ℋ_1_ and ℋ_2_, respectively). Take labeled points *z*_*i*_=(*x*_*i*_, *y*_*i*_) ∈ *𝒳* × *𝒴*, *i* ∈ *ℕ*_*m*_, and unlabeled points *x*_*m*+*i*_ ∈ *𝒳*, *i* ∈ *ℕ*_*u*_. The coregularized least squares algorithm discussed in [11] can be described as follows:

(12) $$\begin{array}{c} \left(f_{z}^{1},f_{z}^{2}\right)≜\arg\underset{f^{2}\in {ℋ}_{2}}{\underset{f^{1}\in {ℋ}_{1}}{\min}}\left\{\frac{1}{m}\underset{i\in N_{m}}{\sum }\ell \left(f^{1}\left(x_{i}\right),f^{2}\left(x_{i}\right),y_{i}\right)+\gamma_{1}{\left|\left|f^{1}\right|\right|}_{{ℋ}_{1}}^{2}+\gamma_{2}{\left|\left|f^{2}\right|\right|}_{{ℋ}_{2}}^{2}+\lambda \overset{m+u}{\underset{i=m+1}{\sum }}{\left(f^{1}\left(x_{i}\right)-f^{2}\left(x_{i}\right)\right)}^{2}\right\}, \end{array}$$

where *m* represents the amount of the labeled points (*x*_*i*_, *y*_*i*_) ∈ *𝒳* × *𝒴*, *i* ∈ *ℕ*_*m*_, and *u* represents the amount of the unlabeled points *x*_*m*+*i*_ ∈ *𝒳*, *i* ∈ *ℕ*_*u*_. The signs *γ*_1_, *γ*_2_, and *λ* mean the regularization parameters. The functions *f*^1^ and *f*^2^, respectively, represent two viewers, and the function *ℓ*(*f*^1^(·), *f*^2^(·), ·) is the labeled loss function, which measures the performance of *f*^1^ and *f*^2^ on the labeled points *z*_*i*_, *i* ∈ *ℕ*_*m*_.

In [11], the final output is denoted as (*f*_**z**_^1^+*f*_**z**_^2^)/2.

In [2], Ying and Campbell applied the Rademacher chaos complexity to study the generalization of multiple kernel learning in the supervised learning framework. The multiple kernel learning model they considered is as follows:

(13) $$\begin{array}{c} f_{z}≜\arg\underset{K\in K}{\min}\underset{f\in {ℋ}_{K}}{\min}\left\{\frac{1}{m}\underset{i\in N_{m}}{\sum }\ell \left(f\left(x_{i}\right),y_{i}\right)+\gamma {\left|\left|f\right|\right|}_{{ℋ}_{K}}^{2}\right\}, \end{array}$$

where *m* represents the amount of the labeled points (*x*_*i*_, *y*_*i*_) ∈ *𝒳* × *𝒴*, *i* ∈ *ℕ*_*m*_. The function *ℓ*(*f*(*x*_*i*_), *y*_*i*_) is the loss function. The sign *λ* means the regularization parameters. And ℋ_*K*_ denotes the reproducing kernel Hilbert spaces. In Equation (13), ℋ_*K*_ is not predefined and depends on the kernel choose from the class *𝒦* of kernel.

In this paper, we are interested in the topic of coregularized multiple kernel learning; that is, the two reproducing kernel Hilbert spaces are not defined in advance. Our discussions are in the framework of semisupervised multiview learning. We give this learning question as the following two-layer minimization formation:

(14) $$\begin{array}{c} \left(f_{z}^{1},f_{z}^{2}\right)≜\arg\underset{K_{2}\in K^{\prime }}{\underset{K_{1}\in K}{\min}}\underset{f^{2}\in {ℋ}_{K_{2}}}{\underset{f^{1}\in {ℋ}_{K_{1}}}{\min}}\left\{\frac{1}{m}\underset{i\in N_{m}}{\sum }\ell \left(f^{1}\left(x_{i}\right),f^{2}\left(x_{i}\right),y_{i}\right)+\gamma_{1}{\left|\left|f^{1}\right|\right|}_{{ℋ}_{K_{1}}}^{2}+\gamma_{2}{\left|\left|f^{2}\right|\right|}_{{ℋ}_{K_{2}}}^{2}+\lambda \overset{m+u}{\underset{i=m+1}{\sum }}{\left(f^{1}\left(x_{i}\right)-f^{2}\left(x_{i}\right)\right)}^{2}\right\}, \end{array}$$

where let *𝒦* and *𝒦*′ be the classes of kernels. ℋ_*K*_1__ and ℋ_*K*_2__ denote the reproducing kernel Hilbert spaces. *m* represents the amount of the labeled points (*x*_*i*_, *y*_*i*_) ∈ *𝒳* × *𝒴*, *i* ∈ *ℕ*_*m*_, and *u* represents the amount of the unlabeled points *x*_*m*+*i*_ ∈ *𝒳*, *i* ∈ *ℕ*_*u*_. The signs *γ*_1_, *γ*_2_, and *λ* mean the regularization parameters. The functions *f*^1^ and *f*^2^, respectively, represent two viewers, and the function *ℓ*(*f*^1^(·), *f*^2^(·), ·) is the labeled loss function, which measures the performance of *f*^1^ and *f*^2^ on the labeled points *z*_*i*_, *i* ∈ *ℕ*_*m*_.

***Remark 2*.**

We will explain the following:

1. Equation (14) given in this paper is different from Equation (12):
  - The solution from Equation (14) is to minimize on the class (∪_*K*_1_∈*𝒦*_ℋ_*K*_1__) × (∪_*K*_2_∈*𝒦*′_ℋ_*K*_2__), while the solution from Equation (12) is to minimize on the class ℋ_*K*_1__ × ℋ_*K*_2__.
  - The solution from Equation (14) is through two minimization steps: first, it finds the most suitable reproducing kernel for the given samples. Second, it obtains the best function/model from the found reproducing kernel Hilbert spaces in the first step. While in Equation (12), it only needs to get the best function/model from the reproducing kernel Hilbert spaces which are predefined.
2. Equation (14) given in this paper is different from Equation (13):
  - The solution from Equation (14) is to minimize on the product space (∪_*K*_1_∈*𝒦*_ℋ_*K*_1__) × (∪_*K*_2_∈*𝒦*′_ℋ_*K*_2__), while the solution from Equation (12) is to minimize on the space (∪_*K*_1_∈*𝒦*_ℋ_*K*_1__).
  - The minimization item in Equation (13) is much simpler because the analysis on Equation (13) is limited to supervised learning and single view and does not deal with unlabeled samples.

From the above discussion, we can see that the generalization analysis about Equation (14) will make more meaningful and bring greater challenge.

In the next section, we will present the main results of this paper.

## 4. Generalization Bounds

In this section, we assume that the loss function *ℓ* in Equation (11) is the subnormalized functional with degenerate dimension 2 on ℋ_*K*_1__ × ℋ_*K*_2__ × *𝒴*, *K*_1_ ∈ *𝒦*, *K*_2_ ∈ *𝒦*′. In Equation (11), let *f*^1^=0 and *f*^1^=0, we have

(15) $$\begin{array}{c} \underset{K_{2}\in K^{\prime }}{\underset{K_{1}\in K}{\min}}\underset{f^{2}\in {ℋ}_{K_{2}}}{\underset{f^{1}\in {ℋ}_{K_{1}}}{\min}}Q\left(f^{1},f^{2}\right)\leq Q\left(0,0\right)=\frac{1}{m}\underset{i\in N_{m}}{\sum }\ell \left(0,0,y_{i}\right)\leq 1, \end{array}$$

Note that 𝒬(*f*^1^, *f*^2^) ≥ 0, and then for any samples (*x*_*i*_, *y*_*i*_), *i* ∈ *ℕ*_*m*_ and *x*_*m*+*i*_, *i* ∈ *ℕ*_*u*_, the class of candidate reproducing kernel Hilbert spaces is defined as follows:

(16) $$\begin{array}{c} \left(f_{z}^{1},f_{z}^{2}\right)\in {ℋ}_{K,K^{\prime }}≜\left\{\left(f^{1},f^{2}\right):\gamma_{1}{\left|\left|f^{1}\right|\right|}_{{ℋ}_{K_{1}}}^{2}+\gamma_{2}{\left|\left|f^{2}\right|\right|}_{{ℋ}_{K_{2}}}^{2}+\lambda \overset{m+u}{\underset{i=m+1}{\sum }}{\left(f^{1}\left(x_{i}\right)-f^{2}\left(x_{i}\right)\right)}^{2}\leq 1,K_{1}\in K,K_{2}\in K^{\prime }\right\}. \end{array}$$

That is, the solution (*f*_**z**_^1^, *f*_**z**_^2^) minimizing 𝒬(*f*^1^, *f*^2^) belongs to the class ℋ_*𝒦*_ × ℋ_*𝒦*′_. For simplicity, we use ℋ_*𝒦*,*𝒦*′_ to denote ℋ_*𝒦*_ × ℋ_*𝒦*′_ in the next sections.

As the assumption in [11], the final predictor for the coregularized multiple kernel learning is selected from the following class:

(17) $$\begin{array}{c} {\hat{ℋ}}_{K,K^{\prime }}≜\left\{\frac{f+g}{2}:\left(f,g\right)\in {ℋ}_{K,K^{\prime }}\right\}. \end{array}$$

***Remark 3*.**

In Equations (16) and (17), we can see that ℋ_*𝒦*,*𝒦*′_ and ${\hat{ℋ}}_{𝒦,{𝒦}^{\prime }}$ mainly depend on the prescribed set of candidate kernels and the unlabeled data.

For any $f\in {\hat{ℋ}}_{𝒦,{𝒦}^{\prime }}$, we define the expected loss as Equation (2), and we can use the loss function *L* to compute the labeled empirical loss in Equation (11). For the given samples (*x*_*i*_, *y*_*i*_) ∈ *𝒳* × *𝒴*, *i* ∈ *ℕ*_*m*_, the loss can be also computed as Equation (3).

If *L* satisfies the Lipschitz continuous condition on ${\hat{ℋ}}_{𝒦,{𝒦}^{\prime }}$, we introduce the constant defined by

(18) $$\begin{array}{c} L_{\sup}=\sup\left\{\left|L\left(f\left(\cdot \right),\cdot \right)\right|:\text{for any}\;\;\left|f\right|\leq \frac{\kappa +\kappa^{\prime }}{\sqrt{\min\left\{\gamma_{1},\gamma_{2}\right\}}}\right\}, \end{array}$$

and the local Lipschitz constant denoted as

(19) $$\begin{array}{c} L_{\sup}^{\text{loc}}=\sup\left\{\frac{\left|L\left(f\left(\cdot \right),\cdot \right)\right|\left|-L\left(f^{\prime }\left(\cdot \right),\cdot \right)\right|}{\left|f-f^{\prime }\right|}:\text{for any}\;\;\left|f\right|,\left|f^{\prime }\right|\leq \frac{\kappa +\kappa^{\prime }}{\sqrt{\min\left\{\gamma_{1},\gamma_{2}\right\}}}\right\}. \end{array}$$

In the end of this section, we give the main theorems in this paper.

**Theorem 1 .**

Let the function L be a subnormalized functional with degenerate dimension 1 on ${\hat{ℋ}}_{𝒦,{𝒦}^{\prime }}\times 𝒴$ and satisfy the Lipschitz continuous condition on ${\hat{ℋ}}_{𝒦,{𝒦}^{\prime }}$. Let the labeled samples *z*_*i*_=(*x*_*i*_, *y*_*i*_) ∈ *𝒳* × *𝒴*, *i* ∈ *ℕ*_*m*_, be independent random variables drawn from the probability space (*𝒳* × *𝒴*, P), and the unlabeled samples *x*_*m*+*i*_ ∈ *𝒳*, *i* ∈ *ℕ*_*u*_, be independent random variables drawn from the probability space (*𝒳*, P_*𝒳*_). Then, for any *δ* ∈ (0,1), with probability at least 1 − *δ*, for any $f\in {\hat{ℋ}}_{𝒦,{𝒦}^{\prime }}$, the following inequality holds

(20) $$\begin{array}{c} E_{\text{P}}\left[L\left(f\left(X\right),Y\right)\right]-{\text{E}}_{n}\left(f,S\right)\leq \frac{L_{\sup}^{\text{loc}}}{\sqrt{m}}{\left\{\frac{2}{\gamma_{1}}U_{m}\left(K\right)+\frac{2}{\gamma_{2}}U_{m}\left(K^{\prime }\right)+\frac{\kappa^{2}}{\gamma_{1}}+\frac{\kappa^{\prime 2}}{\gamma_{2}}+-\frac{1}{\sqrt{m}}\cdot E_{\sigma }\underset{K^{2}\in K^{\prime }}{\underset{K^{1}\in K}{\inf}}\sigma^{T}\cdot {\left(\left(\frac{1}{\gamma_{1}}K_{MU}^{1}-\frac{1}{\gamma_{2}}K_{MU}^{2}\right)\cdot O\cdot \Delta_{UU}\left(\lambda ,K_{1},K_{2}\right)\cdot O^{T}\right)}^{2}\cdot \sigma \right\}}^{1/2}+3L_{\sup}\cdot \sqrt{\frac{\ln\left(2/\delta \right)}{2m}}, \end{array}$$

where **σ** presents a vector (*σ*_1_, *σ*_2_,…, *σ*_*n*_) and *σ*_*i*_, *i* ∈ *ℕ*_*m*_, are Rademacher variables, (1/*γ*_1_) · *K*_*UU*_^1^+(1/*γ*_2_) · *K*_*UU*_^2^=*O* · *D*_*UU*_(*K*_1_, *K*_2_) · *O*^**T**^ with diagonal elements *D*_*ii*_(*K*_1_, *K*_2_) ≥ 0, *i* ∈ *ℕ*_*u*_, and orthogonal matrix O, and

(21) $$\begin{array}{c} D_{UU}\left(K_{1},K_{2}\right)=\left(\begin{array}{cccc} d_{11}\left(K_{1},K_{2}\right) & & & \\ & d_{22}\left(K_{1},K_{2}\right) & & \\ & & \ddots & \\ & & & d_{uu}\left(K_{1},K_{2}\right) \end{array}\right), \end{array}$$

(22) $$\begin{array}{c} \Delta_{UU}\left(\lambda ,K_{1},K_{2}\right)=\left(\begin{array}{cccc} \sqrt{\frac{1}{\left(\lambda^{-1}+d_{11}\left(K_{1},K_{2}\right)\right)}} & & & \\ & \sqrt{\frac{1}{\left(\lambda^{-1}+d_{22}\left(K_{1},K_{2}\right)\right)}} & & \\ & & \ddots & \\ & & & \sqrt{\frac{1}{\left(\lambda^{-1}+d_{uu}\left(K_{1},K_{2}\right)\right)}} \end{array}\right). \end{array}$$

**Corollary 1 .**

Under the assumption of Theorem 1, we have the following inequality:

(23) $$\begin{array}{c} E_{\text{P}}\left[L\left(f\left(X\right),Y\right)\right]-\frac{1}{m}\underset{i\in N_{m}}{\sum }L\left(f\left(x_{i}\right),y_{i}\right)\leq \frac{L_{\sup}^{\text{loc}}}{\sqrt{m}}\left\{\frac{2}{\gamma_{1}}U_{m}\left(K\right)+\frac{2}{\gamma_{2}}U_{m}\left(K^{\prime }\right)+\frac{\kappa^{2}}{\gamma_{1}}+\frac{\kappa^{\prime 2}}{\gamma_{2}}+-\frac{1}{\sqrt{m}}\cdot E_{\sigma }\underset{K^{2}\in K^{\prime }}{\underset{K^{1}\in K}{\inf}}\sigma^{T}\cdot {\left(\left(\frac{1}{\gamma_{1}}K_{MU}^{1}-\frac{1}{\gamma_{2}}K_{MU}^{2}\right)\cdot O\cdot \Delta_{UU}\left(\lambda ,\kappa ,\kappa^{\prime }\right)\cdot O^{T}\right)}^{2}\cdot \sigma \right\}+3L_{\sup}\cdot \sqrt{\frac{\ln\left(2/\delta \right)}{2m}}. \end{array}$$

**Corollary 2 .**

Under the assumption of Theorem 1 and assume *𝒦*=*𝒦*′ and *γ*_1_=*γ*_2_, the following inequality holds

(24) $$\begin{array}{c} E_{\text{P}}\left[L\left(f\left(X\right),Y\right)\right]-\frac{1}{m}\underset{i\in N_{m}}{\sum }L\left(f\left(x_{i}\right),y_{i}\right)\leq \frac{L_{\sup}^{\text{loc}}}{\sqrt{m}}\sqrt{\frac{4}{\gamma_{1}}U_{m}\left(K\right)+2\frac{\kappa^{2}}{\gamma_{1}}}+3L_{\sup}\cdot \sqrt{\frac{\ln\left(2/\delta \right)}{2m}}. \end{array}$$

***Remark 4*.**

We will proof Theorem 1 and Corollaries 1 and 2 in Section 5. And in Section 6, we will reveal that Theorem 1 and Corollary 1 are the extensions of the results in [2] and [11], respectively.

## 5. Proofs

In this section, we will prove Theorem 1 and Corollaries 1 and 2 in Section 4. As the preparation for the next proof, we give two following lemmas (Lemmas 2 and 3) in advance.

**Lemma 2 .**

Let the function L be a subnormalized functional with degenerate dimension 1 on ${\hat{ℋ}}_{𝒦,{𝒦}^{\prime }}\times 𝒴$, and satisfy the Lipschitz continuous condition on ${\hat{ℋ}}_{𝒦,{𝒦}^{\prime }}$. Let the labeled samples *z*_*i*_, *i* ∈ *ℕ*_*m*_, be independently drawn from the (*𝒳* × *𝒴*, P) and the unlabeled samples *x*_*m*+*i*_ ∈ *𝒳*, *i* ∈ *ℕ*_*u*_, be independently from the probability space (*𝒳*, P_*𝒳*_). Then, for any *δ* ∈ (0,1), with probability at least 1 − *δ*, we have the following inequality:

(25) $$\begin{array}{c} \underset{f\in {\hat{ℋ}}_{K,K^{\prime }}}{\sup}\left\{E_{\text{P}}\left[L\left(f\left(X\right),Y\right)\right]-E_{n}\left(f,S\right)\right\}\leq 2E_{\sigma }\left\{\underset{f\in {\hat{ℋ}}_{K,K^{\prime }}}{\sup}\left\{\frac{1}{m}\underset{i\in N_{m}}{\sum }\sigma_{i}\cdot L\left(f\left(x_{i}\right),y_{i}\right)\right\}\right\}+\;3L_{\sup}\cdot \sqrt{\frac{\ln\left(2/\delta \right)}{2m}}, \end{array}$$

where **σ** presents a vector (*σ*_1_, *σ*_2_,…, *σ*_*n*_) and *σ*_*i*_, *i* ∈ *ℕ*_*m*_, are Rademacher variables.

**Proof**

For simplicity, let

(26) $$\begin{array}{c} \Delta \left(N_{m}\right)≜\underset{f\in {\hat{ℋ}}_{K,K^{\prime }}}{\sup}\left\{E_{\text{P}}\left[L\left(f\left(X\right),Y\right)-\frac{1}{m}\underset{i\in N_{m}}{\sum }L\left(f\left(x_{i}\right),y_{i}\right)\right]\right\}. \end{array}$$

Replacing the *i*th sample (*x*_*i*_, *y*_*i*_) in the labeled samples with (*x*_*i*_′, *y*_*i*_′), we have

(27) $$\begin{array}{c} \Delta \left(N_{m}\right)-\underset{f\in {\hat{ℋ}}_{K,K^{\prime }}}{\sup}\left\{E_{\text{P}}\left[L\left(f\left(X\right),Y\right)\right]-\frac{1}{m}\left(\underset{i\in N_{m}/j}{\sum }L\left(f\left(x_{i}\right),y_{i}\right)+L\left(f\left(x_{j}^{\prime }\right),y_{j}^{\prime }\right)\right)\right\}=\underset{f\in {\hat{ℋ}}_{K,K^{\prime }}}{\sup}\left[\frac{1}{m}\left(L\left(f\left(x_{j}\right),y_{j}\right)-L\left(f\left(x_{j}^{\prime }\right),y_{j}^{\prime }\right)\right)\right]. \end{array}$$

By McDiarmid's inequality (Theorem D.3 on page 372 of [15]), with probability at least 1 − *δ*/2, we have

(28) $$\begin{array}{c} \underset{f\in {\hat{ℋ}}_{K,K^{\prime }}}{\sup}\left\{E_{\text{P}}\left[L\left(f\left(X\right),Y\right)\right]-\frac{1}{m}\underset{i\in N_{m}}{\sum }L\left(f\left(x_{i}\right),y_{i}\right)\right\}\leq E_{\text{P}}\left\{\underset{f\in {\hat{ℋ}}_{K,K^{\prime }}}{\sup}\left\{E_{\text{P}}\left[L\left(f\left(X\right),Y\right)\right]-\frac{1}{m}\underset{i\in N_{m}}{\sum }L\left(f\left(x_{i}\right),y_{i}\right)\right\}\right\}+\;L_{\sup}\cdot \sqrt{\frac{\ln\left(2/\delta \right)}{2m}}. \end{array}$$

By the symmetrization argument (page 36 of [15]), we bound the expectation of the first term on the right-hand side of the above inequality (28) as follows:

(29) $$\begin{array}{c} E_{\text{P}}\left\{\underset{f\in {\hat{ℋ}}_{K,K^{\prime }}}{\sup}\left\{E_{\text{P}}\left[L\left(f\left(X\right),Y\right)\right]-\frac{1}{m}\underset{i\in N_{m}}{\sum }L\left(f\left(x_{i}\right),y_{i}\right)\right\}\right\}\leq 2E_{\text{P}}E_{\sigma }\left\{\underset{f\in {\hat{ℋ}}_{K,K^{\prime }}}{\sup}\left\{\frac{1}{m}\underset{i\in N_{m}}{\sum }\sigma_{i}\cdot L\left(f\left(x_{i}\right),y_{i}\right)\right\}\right\}. \end{array}$$

Again, applying McDiarmid's inequality to the right-hand side of the above inequality (29), with probability at least 1 − *δ*/2, we have

(30) $$\begin{array}{c} E_{\text{P}}E_{\sigma }\left\{\underset{f\in {\hat{ℋ}}_{K,K^{\prime }}}{\sup}\left\{\frac{1}{m}\underset{i\in N_{m}}{\sum }\sigma_{i}\cdot L\left(f\left(x_{i}\right),y_{i}\right)\right\}\right\}\leq E_{\sigma }\left\{\underset{f\in {\hat{ℋ}}_{K,K^{\prime }}}{\sup}\left\{\frac{1}{m}\underset{i\in N_{m}}{\sum }\sigma_{i}\cdot L\left(f\left(x_{i}\right),y_{i}\right)\right\}\right\}+\;L_{\sup}\cdot \sqrt{\frac{\ln\left(2/\delta \right)}{2m}}. \end{array}$$

Combining inequalities (28), (29), and (30) yield with probability at least 1 − *δ*:

(31) $$\begin{array}{c} \underset{f\in {\hat{ℋ}}_{K,K^{\prime }}}{\sup}\left\{E_{\text{P}}\left[L\left(f\left(X\right),Y\right)\right]-\frac{1}{m}\underset{i\in N_{m}}{\sum }L\left(f\left(x_{i}\right),y_{i}\right)\right\}\leq 2E_{\sigma }\left\{\underset{f\in {\hat{ℋ}}_{K,K^{\prime }}}{\sup}\left\{\frac{1}{m}\underset{i\in N_{m}}{\sum }\sigma_{i}\cdot L\left(f\left(x_{i}\right),y_{i}\right)\right\}\right\}+3L_{\sup}\cdot \sqrt{\frac{\ln\left(2/\delta \right)}{2m}}. \end{array}$$

**Lemma 3 .**

Under the assumption of Lemma 2, for any *δ* ∈ (0,1), with probability at least 1 − *δ*, we have the following inequality:

(32) $$\begin{array}{c} E_{\sigma }\left\{\underset{f\in {\hat{ℋ}}_{K,K^{\prime }}}{\sup}\left\{\frac{1}{m}\underset{i\in N_{m}}{\sum }\sigma_{i}\cdot L\left(f\left(x_{i}\right),y_{i}\right)\right\}\right\}\leq L_{\sup}^{\text{loc}}\cdot E_{\sigma }\left\{\underset{f\in {\hat{ℋ}}_{K,K^{\prime }}}{\sup}\left\{\frac{1}{m}\underset{i\in N_{m}}{\sum }\sigma_{i}\cdot f\left(x_{i}\right)\right\}\right\}, \end{array}$$

where, **σ** presents a vector (*σ*_1_, *σ*_2_,…, *σ*_*n*_) and *σ*_*i*_, *i* ∈ *ℕ*_*m*_, are Rademacher variables.

**Proof**

As defined in Equation (19), *L*_sup_^loc^ is the local Lipschitz constant of *L*. And by the contraction property of Rademacher complexity (Lemma 26.9 on page 331 of [16] and Theorem 7 of [17]), the result is as follows.

**Lemma 4 .**

*If Equation* (11) *has a solution, then, for a fixedK*^1^ ∈ *𝒦and a fixedK*^2^ ∈ *𝒦*′*, it has a solution*(*f*_*z*_^1^, *f*_*z*_^2^)*as follows:*

(33) $$\begin{array}{c} f_{z}^{1}=\underset{i\in N_{m+u}}{\sum }\alpha_{i}K^{1}\left(x_{i},\cdot \right)\in \left(\underset{K_{1}\in K}{\cup }{ℒ}_{m+u}^{K^{1}}\right), \end{array}$$

(34) $$\begin{array}{c} f_{z}^{2}=\underset{i\in N_{m+u}}{\sum }\beta_{i}K^{2}\left(x_{i},\cdot \right)\in \left(\underset{K_{2}\in K^{\prime }}{\cup }{ℒ}_{m+u}^{K^{2}}\right), \end{array}$$

for some **α**=(*α*_1_, *α*_2_,…,*α*_*m*+*u*_)^**T**^ ∈ *ℝ*^*m*+*u*^ and *β*=(*β*_1_, *β*_2_,…,*β*_*m*+*u*_)^**T**^ ∈ *ℝ*^*m*+*u*^. That is, the solution belongs to (∪_*K*_1_∈*𝒦*_ℒ_*m*+*u*_^*K*^1^^) × (∪_*K*_2_∈*𝒦*′_ℒ_*m*+*u*_^*K*^2^^).

**Proof**

The result follows in a similar way to Proposition 2.3.1 in [11].

**Lemma 5 .**

Let the labeled samples *z*_*i*_=(*x*_*i*_, *y*_*i*_) ∈ *𝒳* × *𝒴*, *i* ∈ *ℕ*_*m*_, be independent random variables drawn from the probability space (*𝒳* × *𝒴*, P) and the unlabeled samples *x*_*m*+*i*_ ∈ *𝒳*, *i* ∈ *ℕ*_*u*_, be independent random variables drawn from the probability space (*𝒳*, P_*𝒳*_). The following inequality holds

(35) $$\begin{array}{c} E_{\sigma }\left\{\underset{f\in {\hat{ℋ}}_{K,K^{\prime }}}{\sup}\left\{\frac{1}{m}\underset{i\in N_{m}}{\sum }\sigma_{i}\cdot f\left(x_{i}\right)\right\}\right\}\leq \frac{1}{2\sqrt{m}}{\left\{\frac{2}{\gamma_{1}}U_{m}\left(K\right)+\frac{2}{\gamma_{2}}U_{m}\left(K^{\prime }\right)+\frac{\kappa^{2}}{\gamma_{1}}+\frac{\kappa^{\prime 2}}{\gamma_{2}}+-\frac{1}{\sqrt{m}}\cdot E_{\sigma }\underset{K^{2}\in K^{\prime }}{\underset{K^{1}\in K}{\inf}}\sigma^{T}\cdot {\left(\left(\frac{1}{\gamma_{1}}K_{MU}^{1}-\frac{1}{\gamma_{2}}K_{MU}^{2}\right)\cdot O\cdot \Delta_{UU}\left(\lambda ,K_{1},K_{2}\right)\cdot O^{T}\right)}^{2}\cdot \sigma \right\}}^{1/2}, \end{array}$$

where **σ** presents a vector (*σ*_1_, *σ*_2_,…, *σ*_*n*_) and *σ*_*i*_, *i* ∈ *ℕ*_*m*_, are Rademacher variables, (1/*γ*_1_) · *K*_*UU*_^1^+(1/*γ*_2_) · *K*_*UU*_^2^=*O* · *D*_*UU*_(*K*_1_, *K*_2_) · *O*^**T**^ with diagonal elements *D*_*ii*_(*K*_1_, *K*_2_) ≥ 0, *i* ∈ *ℕ*_*u*_, and orthogonal matrix O, and

(36) $$\begin{array}{c} D_{UU}\left(K_{1},K_{2}\right)=\left(\begin{array}{cccc} d_{11}\left(K_{1},K_{2}\right) & & & \\ & d_{22}\left(K_{1},K_{2}\right) & & \\ & & \ddots & \\ & & & d_{uu}\left(K_{1},K_{2}\right) \end{array}\right), \end{array}$$

(37) $$\begin{array}{c} \Delta_{UU}\left(\lambda ,K_{1},K_{2}\right)=\left(\begin{array}{cccc} \sqrt{\frac{1}{\left(\lambda^{-1}+d_{11}\left(K_{1},K_{2}\right)\right)}} & & & \\ & \sqrt{\frac{1}{\left(\lambda^{-1}+d_{22}\left(K_{1},K_{2}\right)\right)}} & & \\ & & \ddots & \\ & & & \sqrt{\frac{1}{\left(\lambda^{-1}+d_{uu}\left(K_{1},K_{2}\right)\right)}} \end{array}\right), \end{array}$$

**Proof**

As defined in Equation (17), we can rewrite the left-hand side of inequality (35) as

(38) $$\begin{array}{c} E_{\sigma }\left\{\underset{f\in {\hat{ℋ}}_{K,K^{\prime }}}{\sup}\left\{\frac{1}{m}\underset{i\in N_{m}}{\sum }\sigma_{i}\cdot f\left(x_{i}\right)\right\}\right\}=E_{\sigma }\left\{\underset{\left(f,g\right)\in {ℋ}_{K,K^{\prime }}}{\sup}\left\{\frac{1}{2m}\underset{i\in N_{m}}{\sum }\sigma_{i}\cdot \left[f\left(x_{i}\right)+g\left(x_{i}\right)\right]\right\}\right\}, \end{array}$$

where the sign ℋ_*𝒦*,*𝒦*′_ is defined in Equation (16).

From the assumptions, we have

(39) $$\begin{array}{c} {ℒ}_{m+u}^{K_{1}}≜\text{span}\left\{K^{1}\left(x_{i}\right):x_{i}\in X,i\in N_{m+u}\right\}\subseteq {ℋ}_{K^{1}},K^{1}\in K, \end{array}$$

(40) $$\begin{array}{c} {ℒ}_{m+u}^{K_{2}}≜\text{span}\left\{K^{2}\left(x_{i}\right):x_{i}\in X,i\in N_{m+u}\right\}\subseteq {ℋ}_{K^{2}},K^{2}\in K^{\prime }. \end{array}$$

By the reproducing property and Lemma 1, for any *K*^1^ ∈ *𝒦* and *K*^2^ ∈ *𝒦*′, *for anyi* ∈ *ℕ*_*m*+*u*_, we have

(41) $$\begin{array}{c} f\left(x_{i}\right)=\left({\text{Proj}}_{{ℒ}_{m+u}^{K_{1}}}\right)f\left(x_{i}\right), \end{array}$$

(42) $$\begin{array}{c} f\left(x_{i}\right)=\left({\text{Proj}}_{{ℒ}_{m+u}^{K_{2}}}\right)f\left(x_{i}\right). \end{array}$$

Combining Equations (39), (40), (41), and (42) yields that

(43) $$\begin{array}{c} E_{\sigma }\left\{\underset{\left(f,g\right)\in {ℋ}_{K,K^{\prime }}}{\sup}\left\{\frac{1}{2m}\underset{i\in N_{m}}{\sum }\sigma_{i}\cdot \left[f\left(x_{i}\right)+g\left(x_{i}\right)\right]\right\}\right\}=E_{\sigma }\underset{K^{2}\in K^{\prime }}{\underset{K^{1}\in K}{\sup}}\left\{\frac{1}{2m}\underset{i\in N_{m}}{\sum }\sigma_{i}\cdot \left[f\left(x_{i}\right)+g\left(x_{i}\right)\right]:\left(f,g\right)\in \left({ℒ}_{m+u}^{K_{1}}\times {ℒ}_{m+u}^{K_{2}}\right)\cap {ℋ}_{K^{1},K^{2}}\right\}, \end{array}$$

where

(44) $$\begin{array}{c} {ℋ}_{K_{1},K_{2}}≜\left\{\left(f,g\right):\gamma_{1}{\left|\left|f\right|\right|}_{{ℋ}_{K_{1}}}^{2}+\gamma_{2}{\left|\left|g\right|\right|}_{{ℋ}_{K_{2}}}^{2}+\lambda \overset{m+u}{\underset{i=m+1}{\sum }}{\left(f\left(x_{i}\right)-g\left(x_{i}\right)\right)}^{2}\leq 1\right\}. \end{array}$$

By Lemma 4, for any *K*_1_ ∈ *𝒦* and *K*_2_ ∈ *𝒦*′, we have (this is similar to Section 5.2.1 converting to Euclidean space in [11])

(45) $$\begin{array}{c} {\left(f\left(x_{1}\right),f\left(x_{2}\right),\ldots ,f\left(x_{m}\right)\right)}^{T}=\left(K_{MU}^{1}K_{MM}^{1}\right)\cdot \alpha ,\\ {\left(g\left(x_{1}\right),g\left(x_{2}\right),\ldots ,g\left(x_{m}\right)\right)}^{T}=\left(K_{MU}^{2}K_{MM}^{2}\right)\cdot \beta ,\\ {\left|\left|f\right|\right|}_{{ℋ}_{K_{1}}}^{2}=\alpha^{T}\cdot K^{1}\cdot \alpha ,\\ {\left|\left|g\right|\right|}_{{ℋ}_{K_{2}}}^{2}=\beta^{T}\cdot K^{2}\cdot \beta ,\\ \overset{m+u}{\underset{i=m+1}{\sum }}{\left(f\left(x_{i}\right)-g\left(x_{i}\right)\right)}^{2}=\left(\alpha^{T}\beta^{T}\right)\cdot \left(\begin{array}{c} K_{UU}^{1}\\ K_{MU}^{1}\\ -K_{UU}^{2}\\ -K_{MU}^{2} \end{array}\right)\cdot \left(K_{UU}^{1}K_{UM}^{1}\left(-K_{UU}^{2}\right)\left(-K_{UM}^{2}\right)\right)\cdot \left(\begin{array}{c} \alpha \\ \beta \end{array}\right), \end{array}$$

where

(46) $$\begin{array}{c} K^{i}={\left(\begin{array}{cc} K_{UU}^{i} & K_{UM}^{i}\\ K_{MU}^{i} & K_{MM}^{i} \end{array}\right)}_{\left(m+u\right)\times \left(m+u\right)},\;i=1,2,\\ K_{UU}^{i}={\left(\begin{array}{ccc} K^{i}\left(x_{m+1},x_{m+1}\right) & \ldots & K^{i}\left(x_{m+1},x_{m+u}\right)\\ K^{i}\left(x_{m+2},x_{m+1}\right) & \ldots & K^{i}\left(x_{m+2},x_{m+u}\right)\\ \vdots & \ddots & \vdots \\ K^{i}\left(x_{m+u},x_{m+1}\right) & \ldots & K^{i}\left(x_{m+u},x_{m+u}\right) \end{array}\right)}_{u\times u},\;i=1,2,\\ K_{UM}^{i}={\left(\begin{array}{ccc} K^{i}\left(x_{m+1},x_{1}\right) & \ldots & K^{i}\left(x_{m+1},x_{m}\right)\\ K^{i}\left(x_{m+2},x_{1}\right) & \ldots & K^{i}\left(x_{m+2},x_{m}\right)\\ \vdots & \ddots & \vdots \\ K^{i}\left(x_{m+u},x_{1}\right) & \ldots & K^{i}\left(x_{m+u},x_{m}\right) \end{array}\right)}_{u\times m},\;i=1,2,\\ K_{MU}^{i}={\left(\begin{array}{ccc} K^{i}\left(x_{1},x_{m+1}\right) & \ldots & K^{i}\left(x_{1},x_{m+u}\right)\\ K^{i}\left(x_{2},x_{m+1}\right) & \ldots & K^{i}\left(x_{2},x_{m+u}\right)\\ \vdots & \ddots & \vdots \\ K^{i}\left(x_{m},x_{m+1}\right) & \ldots & K^{i}\left(x_{m},x_{m+u}\right) \end{array}\right)}_{m\times u},\;i=1,2,\\ K_{MM}^{i}={\left(\begin{array}{ccc} K^{i}\left(x_{1},x_{1}\right) & \ldots & K^{i}\left(x_{1},x_{m}\right)\\ K^{i}\left(x_{2},x_{1}\right) & \ldots & K^{i}\left(x_{2},x_{m}\right)\\ \vdots & \ddots & \vdots \\ K^{i}\left(x_{m},x_{1}\right) & \ldots & K^{i}\left(x_{m},x_{m}\right) \end{array}\right)}_{m\times m},\;i=1,2. \end{array}$$

Hence, we have

(47) $$\begin{array}{c} E_{\sigma }\left\{\underset{\left(f,g\right)\in {ℋ}_{K,K^{\prime }}}{\sup}\left\{\frac{1}{2m}\underset{i\in N_{m}}{\sum }\sigma_{i}\cdot \left[f\left(x_{i}\right)+g\left(x_{i}\right)\right]\right\}\right\}=E_{\sigma }\underset{K^{2}\in K^{\prime }}{\underset{K^{1}\in K}{\sup}}\left\{\frac{1}{2m}\underset{i\in N_{m}}{\sum }\sigma_{i}\cdot \left[f\left(x_{i}\right)+g\left(x_{i}\right)\right]:\left(f,g\right)\in \left({ℒ}_{m+u}^{K_{1}}\times {ℒ}_{m+u}^{K_{2}}\right)\;\cap \;{ℋ}_{K^{1},K^{2}}\right\}\\ =E_{\sigma }\underset{K^{2}\in K^{\prime }}{\underset{K^{1}\in K}{\sup}}\underset{\beta \in R^{m+u}}{\underset{\alpha \in R^{m+u}}{\sup}}\left\{\frac{\sigma^{T}\cdot \left(K_{MU}^{1}K_{MM}^{1}\right)\cdot \alpha +\sigma^{T}\cdot \left(K_{MU}^{1}\;\;K_{MM}^{1}\right)\cdot \beta }{2m}:\left(\alpha^{T}\;\;\beta^{T}\right)\cdot \Lambda \cdot \left(\begin{array}{c} \alpha \\ \beta \end{array}\right)\leq 1\right\}, \end{array}$$

where

(48) $$\begin{array}{c} \Lambda =\left(\begin{array}{cc} \gamma_{1}K^{1} & 0\\ 0 & \gamma_{2}K^{2} \end{array}\right)+\lambda \cdot \left(\begin{array}{c} K_{UU}^{1}\\ K_{MU}^{1}\\ -K_{UU}^{2}\\ -K_{MU}^{2} \end{array}\right)\cdot \left(K_{UU}^{1}K_{UM}^{1}\left(-K_{UU}^{2}\right)\left(-K_{UM}^{2}\right)\right). \end{array}$$

Note that the matrix Λ is not full rank, and by using the similar steps in [11], we can rewrite Equation (47) as

(49) $$\begin{array}{c} E_{\sigma }\left\{\underset{\left(f,g\right)\in {ℋ}_{K,K^{\prime }}}{\sup}\left\{\frac{1}{2m}\underset{i\in N_{m}}{\sum }\sigma_{i}\cdot \left[f\left(x_{i}\right)+g\left(x_{i}\right)\right]\right\}\right\}=\frac{1}{2m}E_{\sigma }\underset{K^{2}\in K^{\prime }}{\underset{K^{1}\in K}{\sup}}\left|\left|{\hat{\Lambda \left(K^{1},K^{2}\right)}}^{-1/2}\cdot {\hat{K\left(K^{1},K^{2}\right)}}^{T}\cdot \sigma \right|\right|, \end{array}$$

where

(50) $$\begin{array}{c} \hat{\Lambda \left(K^{1},K^{2}\right)}=\left(K_{MU}^{1}\;\;K_{MM}^{1}\;\;K_{MU}^{2}\;\;K_{MM}^{2}\right)\cdot \left(\begin{array}{cc} V & 0\\ 0 & W \end{array}\right),\\ \hat{K\left(K^{1},K^{2}\right)}=\left(\begin{array}{cc} \gamma_{1}E_{K_{1}} & 0\\ 0 & \gamma_{2}E_{K_{2}} \end{array}\right)+\lambda \cdot R\cdot R^{T},\\ R=\left(\begin{array}{cc} V^{T} & 0\\ 0 & W^{T} \end{array}\right)\cdot \left(\begin{array}{c} K_{UU}^{1}\\ K_{MU}^{1}\\ -K_{UU}^{2}\\ -K_{MU}^{2} \end{array}\right),\\ V^{T}K^{1}V=E_{K_{1}},\\ W^{T}K^{2}W=E_{K_{2}}, \end{array}$$

In the above equations, the matrices *E*_*K*_1__ and *E*_*K*_2__ are, respectively, diagonal matrices containing nonzero eigenvalues. And we write the projections of **α** and **β** onto the column spaces of *K*^1^ and *K*^2^ as *V* · **a** and *W* · **b**.

Next, we try to relate Equation (47) to Rademacher Chaos complexity. Note that

(51) $$\begin{array}{c} \hat{K\left(K^{1},K^{2}\right)}\cdot {\hat{\Lambda \left(K^{1},K^{2}\right)}}^{-1}\cdot {\hat{K\left(K^{1},K^{2}\right)}}^{T}=\frac{1}{\gamma_{1}}K_{MM}^{1}+\frac{1}{\gamma_{2}}K_{MM}^{2}-\lambda \left\{\left(\frac{1}{\gamma_{1}}K_{MU}^{1}-\frac{1}{\gamma_{2}}K_{MU}^{2}\right)\right.\cdot \left.{\left(I+\lambda \left(\frac{1}{\gamma_{1}}K_{UU}^{1}+\frac{1}{\gamma_{2}}K_{UU}^{2}\right)\right)}^{-1}\cdot \left(\frac{1}{\gamma_{1}}K_{UM}^{1}-\frac{1}{\gamma_{2}}K_{UM}^{2}\right)\right\}\\ =\frac{1}{\gamma_{1}}K_{MM}^{1}+\frac{1}{\gamma_{2}}K_{MM}^{2}-{\left(\left(\frac{1}{\gamma_{1}}K_{MU}^{1}-\frac{1}{\gamma_{2}}K_{MU}^{2}\right)\cdot O\cdot \Delta_{UU}\left(\lambda ,K_{1},K_{2}\right)\cdot O^{T}\right)}^{2}, \end{array}$$

The first equation can be easily obtained by the discussion of Section 5.2.4 in [11]. The second equation from

(52) $$\begin{array}{c} \lambda {\left(I+\lambda \left(\frac{1}{\gamma_{1}}K_{UU}^{1}+\frac{1}{\gamma_{2}}K_{UU}^{2}\right)\right)}^{-1}=O\cdot {\left(\lambda^{-1}\cdot I+D_{UU}\left(K_{1},K_{2}\right)\right)}^{-1}\cdot O^{T}\\ =O\cdot {\left(\begin{array}{cccc} \lambda^{-1}+d_{11}\left(K_{1},K_{2}\right) & & & \\ & \lambda^{-1}+d_{22}\left(K_{1},K_{2}\right) & & \\ & & \ddots & \\ & & & \lambda^{-1}+d_{UU}\left(K_{1},K_{2}\right) \end{array}\right)}^{-1}\cdot O^{T}\\ ={\left(O\cdot \left(\begin{array}{cccc} \sqrt{\frac{1}{\left(\lambda^{-1}+d_{11}\left(K_{1},K_{2}\right)\right)}} & & & \\ & \sqrt{\frac{1}{\left(\lambda^{-1}+d_{22}\left(K_{1},K_{2}\right)\right)}} & & \\ & & \ddots & \\ & & & \sqrt{\frac{1}{\left(\lambda^{-1}+d_{uu}\left(K_{1},K_{2}\right)\right)}} \end{array}\right)\cdot O^{T}\right)}^{2} \end{array}$$

Then, we have

(53) $$\begin{array}{c} E_{\sigma }\underset{K^{2}\in K^{\prime }}{\underset{K^{1}\in K}{\sup}}\left|\left|{\hat{\Lambda \left(K^{1},K^{2}\right)}}^{-1/2}\cdot {\hat{K\left(K^{1},K^{2}\right)}}^{T}\cdot \sigma \right|\right|\\ =E_{\sigma }\underset{K^{2}\in K^{\prime }}{\underset{K^{1}\in K}{\sup}}\left\{\sqrt{\sigma^{T}\cdot \frac{1}{\gamma_{1}}K_{MM}^{1}\cdot \sigma +\sigma^{T}\cdot \frac{1}{\gamma_{2}}K_{MM}^{2}\cdot \sigma }\right.\\ =E_{\sigma }\underset{K^{2}\in K^{\prime }}{\underset{K^{1}\in K}{\sup}}\left\{\sigma^{T}\cdot \frac{1}{\gamma_{1}}K_{MM}^{1}\cdot \sigma +\sigma^{T}\cdot \frac{1}{\gamma_{2}}K_{MM}^{2}\cdot \sigma -\sigma^{T}\cdot {\left(\left(\frac{1}{\gamma_{1}}K_{MU}^{1}-\frac{1}{\gamma_{2}}K_{MU}^{2}\right)\cdot O\cdot \Delta_{UU}\left(\lambda ,K_{1},K_{2}\right)\cdot O^{T}\right)}^{2}\right\}\cdot \\ =E_{\sigma }\underset{K^{2}\in K^{\prime }}{\underset{K^{1}\in K}{\sup}}\left\{\frac{1}{\gamma_{1}}\underset{i,j\in N_{m}}{\sum }\sigma_{i}\cdot \sigma_{i}\cdot K^{1}\left(x_{i},x_{j}\right)+\frac{1}{\gamma_{2}}\underset{i,j\in N_{m}}{\sum }\sigma_{i}\cdot \sigma_{i}\cdot K^{2}\left(x_{i},x_{j}\right)\right.-{\left.\sigma^{T}\cdot {\left(\left(\frac{1}{\gamma_{1}}K_{MU}^{1}-\frac{1}{\gamma_{2}}K_{MU}^{2}\right)\cdot O\cdot \Delta_{UU}\left(\lambda ,K_{1},K_{2}\right)\cdot O^{T}\right)}^{2}\cdot \sigma \right\}}^{1/2}\\ \leq E_{\sigma }\underset{K^{2}\in K^{\prime }}{\underset{K^{1}\in K}{\sup}}\left\{\frac{2}{\gamma_{1}}\underset{i,j\in N_{m},i<j}{\sum }\sigma_{i}\cdot \sigma_{j}\cdot K^{1}\left(x_{i},x_{j}\right)+\frac{2}{\gamma_{2}}\underset{i,j\in N_{m},i<j}{\sum }\sigma_{i}\cdot \sigma_{j}\cdot K^{2}\left(x_{i},x_{j}\right)\right.+\frac{1}{\gamma_{1}}\underset{i\in N_{m}}{\sum }K^{1}\left(x_{i},x_{i}\right)+\frac{1}{\gamma_{2}}\underset{i\in N_{m}}{\sum }K^{2}\left(x_{i},x_{i}\right)\\ -{\left.\sigma^{T}\cdot {\left(\left(\frac{1}{\gamma_{1}}K_{MU}^{1}-\frac{1}{\gamma_{2}}K_{MU}^{2}\right)\cdot O\cdot \Delta_{UU}\left(\lambda ,K_{1},K_{2}\right)\cdot O^{T}\right)}^{2}\cdot \sigma \right\}}^{1/2}\\ \leq \left\{\frac{2m}{\gamma_{1}}U_{m}\left(K\right)+\frac{2m}{\gamma_{2}}U_{m}\left(K^{\prime }\right)+\frac{\kappa^{2}m}{\gamma_{1}}+\frac{\kappa \prime^{2}m}{\gamma_{2}}\right.-{\left.E_{\sigma }\underset{K^{2}\in K^{\prime }}{\underset{K^{1}\in K}{\inf}}\sigma^{T}\cdot {\left(\left(\frac{1}{\gamma_{1}}K_{MU}^{1}-\frac{1}{\gamma_{2}}K_{MU}^{2}\right)\cdot O\cdot \Delta_{UU}\left(\lambda ,K_{1},K_{2}\right)\cdot O^{T}\right)}^{2}\cdot \sigma \right\}}^{1/2}. \end{array}$$

The second equation uses Equation (51), the forth inequality is obtained by using Jensen's inequality twice, and the last inequality uses the definition of Rademacher chaos complexity and the finite of kernels.

**Proof**

For any $f\in {\hat{ℋ}}_{𝒦,{𝒦}^{\prime }}$, it is easy to show that

(54) $$\begin{array}{c} E_{\text{P}}\left[L\left(f\left(X\right),Y\right)\right]-\underset{i\in N_{m}}{\sum }L\left(f\left(x_{i}\right),y_{i}\right)\leq \underset{f\in {\hat{ℋ}}_{K,K^{\prime }}}{\sup}\left\{E_{\text{P}}\left[L\left(f\left(X\right),Y\right)\right]-\underset{i\in N_{m}}{\sum }L\left(f\left(x_{i}\right),y_{i}\right)\right\}, \end{array}$$

Combining Lemmas 2, 3, 4, and 5, the result is as follows.

**Proof**

By Gershgorin Circle Theorem (Theorem 7.2.1 on page 381 of [18]), the *D*_*UU*_(*K*_1_, *K*_2_) in Equation (21) can be estimated as follows:

(55) $$\begin{array}{c} d_{ii}\left(K_{1},K_{2}\right)\leq u\cdot \left(\frac{\kappa^{2}}{\gamma_{1}}+\frac{\kappa \prime^{2}}{\gamma_{2}}\right),i\in N_{u}, \end{array}$$

Then, we can write Equation (22) as

(56) $$\begin{array}{c} \Delta_{UU}\left(\lambda ,K_{1},K_{2}\right)\geq \Delta_{UU}\left(\lambda ,\kappa ,\kappa^{\prime }\right)\\ ≜\left(\begin{array}{cccc} \sqrt{\frac{1}{\left(\lambda^{-1}+u\cdot \left(\kappa^{2}/\gamma_{1}+\kappa \prime^{2}/\gamma_{2}\right)\right)}} & & & \\ & \sqrt{\frac{1}{\left(\lambda^{-1}+u\cdot \left(\kappa^{2}/\gamma_{1}+\kappa \prime^{2}/\gamma_{2}\right)\right)}} & & \\ & & \ddots & \\ & & & \sqrt{\frac{1}{\left(\lambda^{-1}+u\cdot \left(\kappa^{2}/\gamma_{1}+\kappa \prime^{2}/\gamma_{2}\right)\right)}} \end{array}\right), \end{array}$$

So, the result is as follows.

**Proof**

By the assumption in Corollary 2, we have

(57) $$\begin{array}{c} \frac{2}{\gamma_{1}}U_{m}\left(K\right)+\frac{2}{\gamma_{2}}U_{m}\left(K^{\prime }\right)+\frac{\kappa^{2}}{\gamma_{1}}+\frac{\kappa^{\prime 2}}{\gamma_{2}}=\frac{4}{\gamma_{1}}U_{m}\left(K\right)+2\frac{\kappa^{2}}{\gamma_{1}}=\frac{4}{\gamma_{2}}U_{m}\left(K^{\prime }\right)+2\frac{\kappa^{\prime 2}}{\gamma_{2}}, \end{array}$$

(58) $$\begin{array}{c} \frac{1}{\sqrt{m}}\cdot E_{\sigma }\underset{K^{2}\in K^{\prime }}{\underset{K^{1}\in K}{\inf}}\sigma^{T}\cdot {\left(\left(\frac{1}{\gamma_{1}}K_{MU}^{1}-\frac{1}{\gamma_{2}}K_{MU}^{2}\right)\cdot O\cdot \Delta_{UU}\left(\lambda ,\kappa ,\kappa^{\prime }\right)\cdot O^{T}\right)}^{2}\cdot \sigma =0. \end{array}$$

By substituting Equations (57) and (58) into Equation (24), the Corollary 2 follows.

## 6. Discussion

In the above two sections, we give our main results and proof these results. In this section, we will give a comparative discussion of our results to the existing work (in [2] and [11]).

First, we can see that the term

(59) $$\begin{array}{c} \frac{1}{\sqrt{m}}\cdot E_{\sigma }\underset{K^{2}\in K^{\prime }}{\underset{K^{1}\in K}{\inf}}\sigma^{T}\cdot {\left(\left(\frac{1}{\gamma_{1}}K_{MU}^{1}-\frac{1}{\gamma_{2}}K_{MU}^{2}\right)\cdot O\cdot \Delta_{UU}\left(\lambda ,K_{1},K_{2}\right)\cdot O^{T}\right)}^{2}\cdot \sigma \end{array}$$

in Theorem 1 reflects the compatibility of two viewers on the unlabeled samples.

1. For multiple kernel learning in supervised learning.

If we let *u*=0 and *γ*_1_=*γ*_2_=*λ*, then we have

(60) $$\begin{array}{c} \begin{array}{cc} & \frac{2}{\gamma_{1}}U_{m}\left(K\right)+\frac{2}{\gamma_{2}}U_{m}\left(K^{\prime }\right)+\frac{\kappa^{2}}{\gamma_{1}}+\frac{\kappa^{\prime 2}}{\gamma_{2}}=\frac{4}{\lambda }U_{m}\left(K\right)+2\frac{\kappa^{2}}{\lambda }, \end{array} \end{array}$$

(61) $$\begin{array}{c} \frac{1}{\sqrt{m}}\cdot E_{\sigma }\underset{K^{2}\in K^{\prime }}{\underset{K^{1}\in K}{\inf}}\sigma^{T}\cdot {\left(\left(\frac{1}{\gamma_{1}}K_{MU}^{1}-\frac{1}{\gamma_{2}}K_{MU}^{2}\right)\cdot O\cdot \Delta_{UU}\left(\lambda ,\kappa ,\kappa^{\prime }\right)\cdot O^{T}\right)}^{2}\cdot \sigma =0. \end{array}$$

By Corollary (1) and Equations (60) and (61), we can get that

(62) $$\begin{array}{c} E_{\text{P}}\left[L\left(f\left(X\right),Y\right)\right]-\frac{1}{m}\underset{i\in N_{m}}{\sum }L\left(f\left(x_{i}\right),y_{i}\right)\leq \frac{L_{\sup}^{\text{loc}}}{\sqrt{m}}{\left\{\frac{4}{\lambda }U_{m}\left(K\right)+2\frac{\kappa^{2}}{\lambda }\right\}}^{1/2}+3L_{\sup}\cdot \sqrt{\frac{\ln\left(2/\delta \right)}{2m}}. \end{array}$$

Then, the main result in [2] recovers from Corollary (1). Therefore, the main result in [2] becomes the special case of Corollary (1).

***Remark 5*.**

For the discussion in Section 3, if we set *u*=0 and *γ*_1_=*γ*_2_=*λ* and by combining Equation (17), then we have that Equation (14) reduces to Equation (13). Furthermore, let |*𝒦*|=|*𝒦*′|=1 and *𝒦*=*𝒦*′, and we have

(63) $$\begin{array}{c} U_{m}\left(K\right)=U_{m}\left(K\right)=\leq \frac{2}{m}\underset{i\in {\mathbb{N}}_{m}}{\sum }K\left(x_{i},x_{i}\right). \end{array}$$

Substituting inequality (63) into inequality (62), we can obtain the generalization bound for the single kernel learning in the framework of supervised learning as follows:

(64) $$\begin{array}{c} E_{\text{P}}\left[L\left(f\left(X\right),Y\right)\right]-\frac{1}{m}\underset{i\in N_{m}}{\sum }L\left(f\left(x_{i}\right),y_{i}\right)\leq \frac{L_{\sup}^{\text{loc}}}{\sqrt{m}}{\left\{\frac{8}{m\lambda }\underset{i\in N_{m}}{\sum }K\left(x_{i},x_{i}\right)+2\frac{\kappa^{2}}{\lambda }\right\}}^{1/2}+3L_{\sup}\cdot \sqrt{\frac{\ln\left(2/\delta \right)}{2m}}. \end{array}$$

(2) For coregularized kernel learning in semisupervised learning.

If we let *K*_1_ ∈ *𝒦*, |*𝒦*|=1, *K*_2_ ∈ *𝒦*′, and |*𝒦*′|=1, by equation we have

(65) $$\begin{array}{c} E_{\sigma }\left\{\underset{\left(f,g\right)\in {ℋ}_{K,K^{\prime }}}{\sup}\left\{\frac{1}{2m}\underset{i\in N_{m}}{\sum }\sigma_{i}\cdot \left[f\left(x_{i}\right)+g\left(x_{i}\right)\right]\right\}\right\}\\ =\frac{1}{2m}E_{\sigma }\underset{K^{2}\in K^{\prime }}{\underset{K^{1}\in K}{\sup}}\left|\left|{\hat{\Lambda \left(K^{1},K^{2}\right)}}^{-1/2}\cdot {\hat{K\left(K^{1},K^{2}\right)}}^{T}\cdot \sigma \right|\right|\\ =\frac{1}{2m}E_{\sigma }\left|\left|{\hat{\Lambda \left(K^{1},K^{2}\right)}}^{-1/2}\cdot {\hat{K\left(K^{1},K^{2}\right)}}^{T}\cdot \sigma \right|\right|. \end{array}$$

And note that Equation (65) is the same as the supremum evaluation in Section 5.2.2 in [11]. So, the main result in [11] recovers from Theorem 1. Then, we have that the main result in [11] can be regarded as the special case of Theorem 1.

***Remark 6*.**

For the discussion in Section 3, if we set *K*_1_ ∈ *𝒦*, |*𝒦*|=1, *K*_2_ ∈ *𝒦*′, and |*𝒦*′|=1, then we have that Equation (14) reduces to Equation (12).

**Figure 1:**
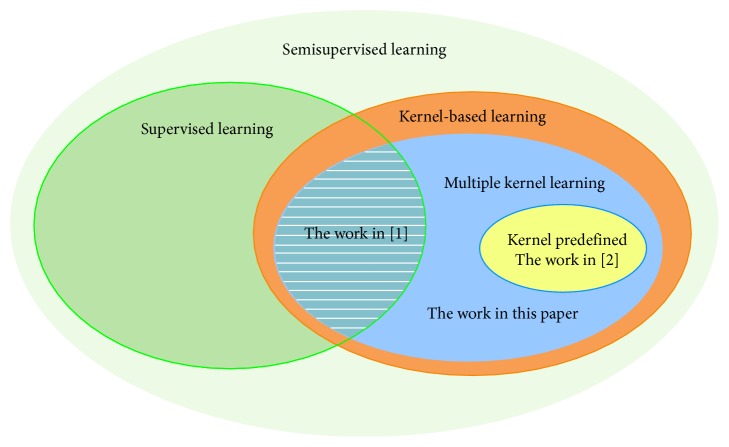
Semisupervised learning as supervised learning when *u*=0. And if *𝒦* has a single kernel, we think that it is the special case of multiple kernel learning. The scope of the discussion in [2] is the intersection of the green and blue ellipses, the scope of the discussion in [11] is the yellow ellipse, and the cope of the discussion in this paper is the blue ellipse.

In Figure 1, we show the relations between the main results in this paper and the results in [2] and [11].

## 7. Conclusion

In this paper, based on semisupervised two-viewers learning, we study the generalization bound of coregularized multiple kernel learning. The main research tool is Rademacher chaos complexity which we use to control the performance of the candidate class of coregularized multiple kernels. In this paper, we mainly blend the work in [2] and [11] to discuss the generalization error of coregularized multiple kernel learning in the semisupervised multiview learning framework. First, we discuss the differences between the learning question proposed by us and the learning questions in [2] and [11]. Then, we analyze the generalization bound of coregularized multiple kernel learning in the semisupervised multiview learning framework. And we show that the existing results in [2] and [11] can be regarded as the special cases of our main results.

## Data Availability

The data used to support the findings of this study are available from the corresponding author upon request.
